# Supplementary material for: Development of a High-Resolution Melting Method for the Detection of Clarithromycin-Resistant Helicobacter pylori in the Gastric Microbiome
Source: Antibiotics (Basel). 2024 Oct 16;13(10):975. doi: 10.3390/antibiotics13100975 (PMC11505316; doi:10.3390/antibiotics13100975)
Supplement: Supplementary file 1 [file antibiotics-13-00975-s001.zip › antibiotics-3171349-supplementary.pdf]

antibiotics

MDPI

Supplementary Materials

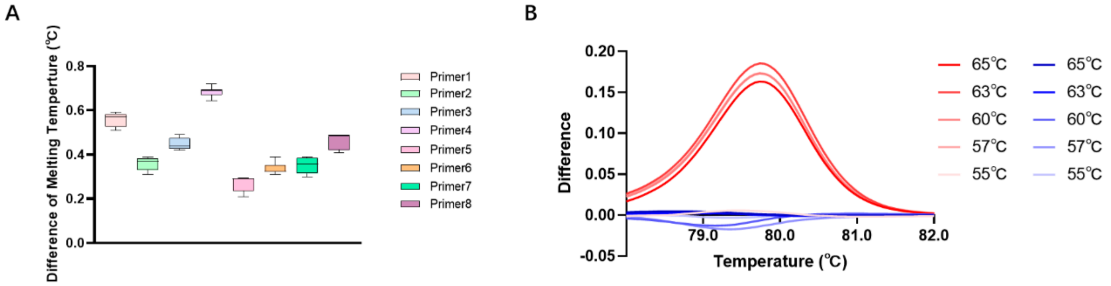

Figure S1. Exploration of HRM primer selection and amplification conditions. (A) Differential Tm values resulting from amplification using 8 different primers, generating amplicon sizes ranging from 50 to 120 bp. We measured the resolution in terms of the temperature difference between A and G bases caused by 8 pairs of different primers. The greater the temperature difference, the higher the resolution. (B) Dissociation curve plot at different extension temperatures for HRM reactions, where the purple gradient curve represents the sample detection curve for the 2143 allele A, and the red gradient curve represents the sample detection curve for the 2143 allele G.

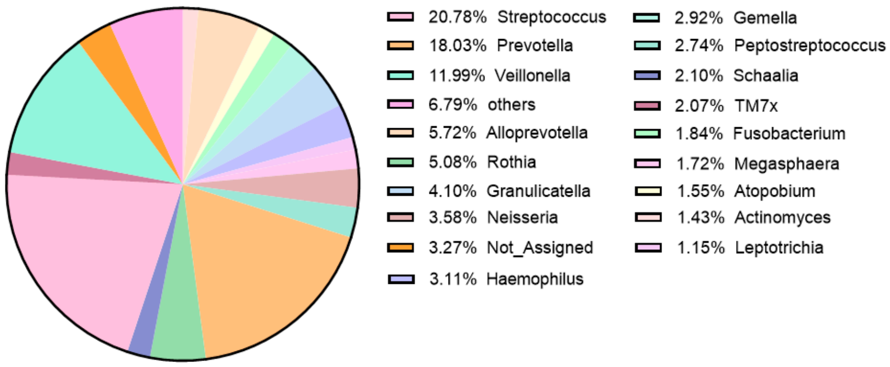

Figure S2. Gastric microbiota profile of a patient from the Shenzhen cohort.

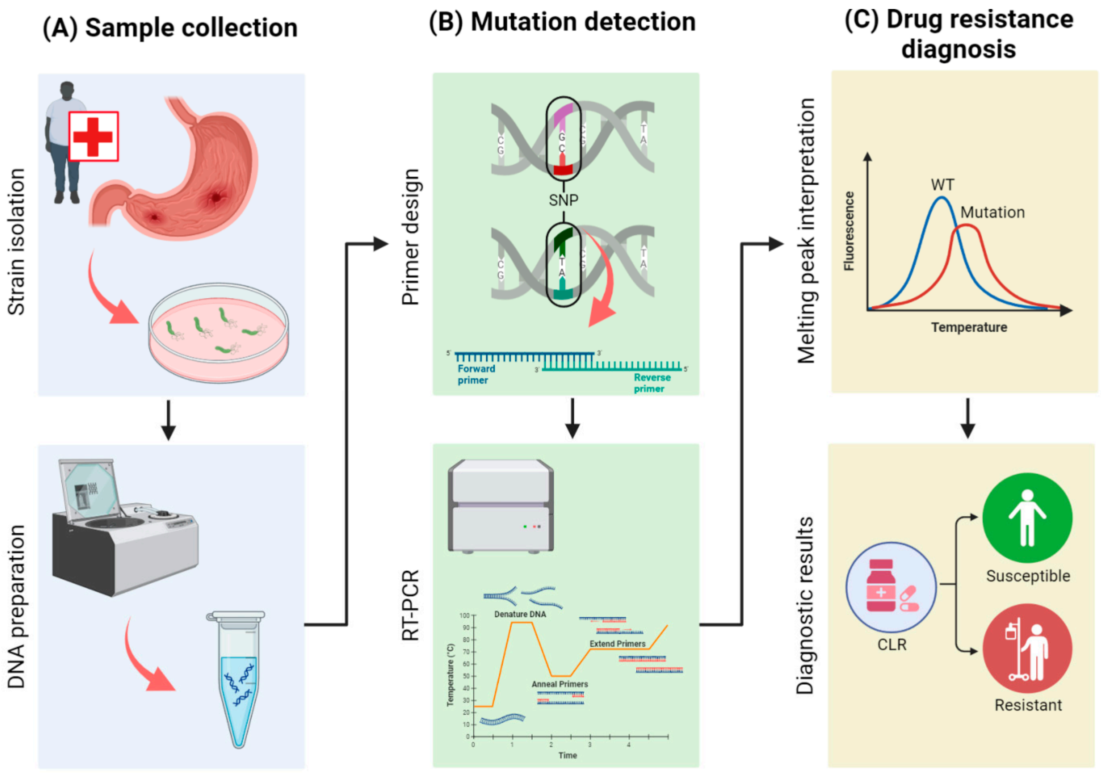

Figure S3. The protocol of HRM. (A) Sample collection. This step primarily involved collecting genomic samples of H. pylori infection from patients to serve as templates for subsequent testing. (B) Mutation detection. This step involved synthesizing primers specific to the SNP sites and conducting RT-PCR amplification on the template DNA. (C) Drug resistance diagnosis. By identifying whether the amplification peak belonged to the WT or mutant, the sensitivity or drug resistance of the sample could be diagnosed.

Table S1. Number of strains for each antibiotic at different MIC values.

Antibiotic	Number of strains									
	<0.125	0.125	0.25	0.5	1	2	4	8	16	>16
CLR			18	2		15				
LVX				15	2	2	16			
AMX	17	2	3	2	5	1	1	4		
MTZ							4	2	7	22
TET				27	5	2	1			

Table S2. The primers used for the *23S rRNA* A2143G mutation target.

**Primer number**	**Sequence (5'→3')**	**Primer length (bp)**	**Product length (bp)**
Primer 1	F1-CTCCTACCCGCGGCAAGAC	19	50
	R1-AAGTTGTAGTAAAGGTCCACGGG	23	
Primer 2	F1-GGTGAAAATTCCTCCTACCCGC	22	60
	R1-AGTTGTAGTAAAGGTCCACGGG	22	
Primer 3	F1-AGGTGAAAATTCCTCCTACCCG	22	70
	R1-GCAGTGCTAAGTTGTAGTAAAGGT	24	
Primer 4	F1-TTGTAGTGGAGGTGAAAATTCCTC	24	80

	R1-AGCAGTGCTAAGTTGTAGTAAAGGT	25	
Primer 5	F1-GAGGTGAAAATTCCTCCTACCC	22	90
	R1-GCGCATGATATTCCCATTAGCAG	23	
Primer 6	F1-TGTAGTGGAGGTGAAAATTCCT	22	101
	R1-TCCTGCGCATGATATTCCCATTA	23	
Primer 7	F1-TTCAGTGAAATTGTAGTGGAGGTG	24	110
	R1-CTGCGCATGATATTCCCATTAGC	23	
Primer 8	F1-CAGTGAAATTGTAGTGGAGGTGA	23	121
	R1-CCTCCCACCTATCCTGCG	18	

Table S3. The standard strains used to assess the specificity of the HRM method.

No.	Type of Standard Strain
1	*Streptococcus anginosus*
2	*Streptococcus parasanguinis*
3	*Streptococcus salivarius*
4	*Streptococcus pseudopneumoniae*
5	*Streptococcus oralis*
6	*Streptococcus sanguinis*
7	*Bacillus nealsonii*
8	*Bacillus aryabhattai*
9	*Bacillus stratosphericus*
10	*Bacillus altitudinis*
11	*Rothia dentocariosa*
12	*Rothia mucilaginosa*
13	*Staphylococcus warneri*
14	*Gemella sanguinis*
